# Memory color influences conscious object perception

**DOI:** 10.1093/nc/niag055

**Published:** 2026-09-02

**Authors:** Vincent Plikat, Pablo R Grassi, Michael M Bannert, Andreas Bartels

**Affiliations:** Vision and Cognition Lab., Department of Psychology, University of Tübingen, Schleichstr. 4, 72076 Tübingen, Germany; Centre for Integrative Neuroscience, Otfried-Müller-Str. 25, 72076 Tübingen, Germany; Max-Planck Institute for Biological Cybernetics, Max-Planck-Ring 8, 72076 Max-Planck-Ring 8, 72076 Tübingen, Germany; Vision and Cognition Lab., Department of Psychology, University of Tübingen, Schleichstr. 4, 72076 Tübingen, Germany; Centre for Integrative Neuroscience, Otfried-Müller-Str. 25, 72076 Tübingen, Germany; Max-Planck Institute for Biological Cybernetics, Max-Planck-Ring 8, 72076 Max-Planck-Ring 8, 72076 Tübingen, Germany; Vision and Cognition Lab., Department of Psychology, University of Tübingen, Schleichstr. 4, 72076 Tübingen, Germany; Centre for Integrative Neuroscience, Otfried-Müller-Str. 25, 72076 Tübingen, Germany; Max-Planck Institute for Biological Cybernetics, Max-Planck-Ring 8, 72076 Max-Planck-Ring 8, 72076 Tübingen, Germany; Vision and Cognition Lab., Department of Psychology, University of Tübingen, Schleichstr. 4, 72076 Tübingen, Germany; Centre for Integrative Neuroscience, Otfried-Müller-Str. 25, 72076 Tübingen, Germany; Max-Planck Institute for Biological Cybernetics, Max-Planck-Ring 8, 72076 Max-Planck-Ring 8, 72076 Tübingen, Germany

**Keywords:** color vision, memory color, predictive coding, top-down effects, cognitive penetrability, ambiguous perception

## Abstract

Can knowledge influence perception? A central case suggesting it can, is evidence showing that knowledge about a color-diagnostic object’s typical color can influence its appearance. For example, a gray banana is allegedly perceived with a tint of yellow. However, methodological and conceptual considerations leave it unclear whether the purported “memory color” effect actually reflects changes in perception or changes in judgment and responses instead. Here, we combine memory color with binocular rivalry to test if top-down influences affect the color an object is perceived in. We showed 24 participants familiar objects in their typical and opponent color and asked for concurrent reports of the perceived color. Consistent with Bayesian models of rivalry, we observed that conscious perception of identical spectral color pairs was biased toward the typical color of the presented object. Our results suggest that prior knowledge aids interpretation of ambiguous stimuli and biases conscious perception toward the most plausible interpretation.

**Highlights**

- Some objects have strong associations to specific colors (e.g. bananas and yellow), their memory colors. We investigated how memory color influences what stimuli enter visual consciousness.
- We employed a binocular rivalry paradigm to measure visual consciousness, thereby minimizing the influence of judgment-related response biases on perceptual report.
- Typically colored objects were more likely to enter consciousness compared to objects in atypical colors.
- We interpret our findings in relation to Bayesian perception theory and to the debate over cognitive penetrability.

## Introduction

Knowledge is said to directly influence perceptual processes (de Lange et al. 2018). For example, when sensory input is noisy and ambiguous, memory-based expectations can be used to aid interpretation and facilitate processing (e.g. when using context to make sense of a spoken word in a noisy environment). Conversely, when sensory input is salient or deviates from expectations, selective attention boosts its processing, prioritizing the unexpected information for the refinement of future predictions.

A prominent example of these influences of cognition in perception relates to memory color (“Gedächtnisfarbe,” Hering 1920). Through experience, we learn that certain objects—termed color-diagnostic—generally have a particular typical color: grass is green, and bananas are yellow. These object-color associations can bias perception of grayscale objects toward their associated color (Hansen et al. 2006, Olkkonen et al. 2008, Witzel et al. 2011, Kimura et al. 2013). In turn, color-diagnostic objects in their incongruent color (e.g. a blue banana) are unexpected and capture attention, e.g. enhancing performance in tasks like change detection (Cutler et al. 2024) or interfering with object recognition (Teichmann et al. 2020). We refer to any influence of prior knowledge about an object’s typical color on perception as the “memory color effect” (MCE). However, the role that memory color plays in determining what color enters conscious perception is unclear. This is particularly relevant given that color vision is subjective and therefore must be studied with tools that dissociate subjective experience from sensory input (Kim et al. 2020).

Moreover, the significance of the MCE extends beyond the field of color vision. It is often regarded as an influential paradigm of cognitive penetrability of perception, i.e. of top-down effects on perception in general. However, it has recently been challenged that the MCE reflects a change in *perception* itself, arguing instead that it influences only (accompanying) cognitive processes like *attention, judgment,* or *recognition* (Firestone and Scholl 2016, Valenti and Firestone 2019, Block 2023), but not how stimuli perceptually appear *per se*. By this account, when participants adjust a banana’s color to make it appear gray and overshoot toward blue chromaticities (Hansen et al. 2006), it is not because they perceive the gray banana with a tint of yellow. Instead, prior knowledge of the banana’s typical yellow color causes subjects to only bias the judgment of their unaffected percepts toward the opponent color (i.e. blue), without changes in visual appearance *per se* (Valenti and Firestone 2019). As it happens, participants are perfectly capable of telling that, for example, an objectively gray banana is the same color as a gray patch and not a yellow patch (Valenti and Firestone 2019). Hence, while MCE can be shown using side-by-side comparisons (Witzel 2016), it is possible that the MCE might not be perceptual after all (Valenti and Firestone 2019).

To address both questions, we combined MCE with binocular rivalry (BR) and tested if memory-based color associations could modulate spontaneous and automatic *conscious perception*. In BR, different images are presented to the two eyes, leading to spontaneous and effortless perceptual alternations despite constant stimulation, allowing to dissociate sensory input from conscious perception (Blake and Logothetis 2002). We presented participants with color-diagnostic objects (e.g. a banana) in a congruent color (yellow) to one eye and an incongruent color (blue) to the other, asking them to report what they concurrently perceived.

We employed BR to: (1) isolate the effect of memory-based color associations on spontaneous and subjective color perception, independent of the spectral properties of the stimulus input and (2) minimize the confounding effect of attention or judgment on the MCE. Notably, in contrast to previous studies investigating memory color (Hansen et al. 2006, Olkkonen et al. 2008, Witzel et al. 2011), we do not aim to measure how color appears perceptually, but rather what enters and dominates visual consciousness in the first place. We hypothesized that a color’s probability to enter visual consciousness would differ depending on whether or not it is congruent or incongruent with object-based expectation. We were neutral with respect to the direction of this difference: on the one hand, previous work found dominance of expected input (Denison et al. 2011, Attarha and Moore 2015) typically interpreted in a Bayesian predictive coding framework as reflecting the higher prior probability of the expectation-based inputs (Hohwy et al. 2008, Brascamp et al. 2018). However, on the other hand further work found dominance of the unexpected input (Mudrik et al. 2011) potentially due to attentional capture and preferential processing. Elucidating which direction applies to memory color was the third aim of our study.

## Materials and methods

### Participants

We measured 24 subjects (10 male, 13 female, one other, 22 right-handed, age 23.2 ± 3.8). This allowed for counterbalanced conditions and detection of effect sizes of *d* = 0.6 with a power of 1 − *β* = 0.8 at *α* = 0.05 for paired t-tests. All subjects had normal or corrected-to-normal vision, and no color vision impairment as assessed with the Ishihara test procedure. Ten of the subjects were not naive to the research question at hand, one of the authors (V.P.) and nine cognitive science bachelor’s students, for whom the experiment was part of a university seminar. All subjects provided written consent prior to the experiments. The study was approved by the ethics committee of the Department of Psychology at the University of Tübingen.

### Apparatus

Stimuli were presented on a linearized monitor at a distance of 70 cm using MATLAB R2023b with Psychtoolbox 3 (Brainard 1997, Kleiner et al. 2007). Subjects viewed the stimuli through a mirror stereoscope and provided their responses using a dedicated keyboard with three keys.

### Experimental rationale, design, and procedure

In this study, we tested the influence of prior knowledge about the typical color of common color-diagnostic objects on conscious color perception during BR. To this end, we created a BR experiment in which we simultaneously presented color-diagnostic objects in their congruent color (e.g. a yellow banana) to one eye and in an incongruent color (e.g. a blue banana) to the other eye. We selected four prototypical colors (red, green, yellow, and blue) from the CIEL^*^a^*^b^*^ color space, which is approximately perceptually uniform. The two pairs of opposing colors, red–green and yellow–blue, were always presented together during rivalry. Importantly, to control low-level chromatic properties, we used the same hues when presenting a color on a congruent or incongruent object, e.g. the congruent red on the tomato was the same as the incongruent red on the lettuce, and *vice versa* for the paired green hue (see section *Stimuli generation and display* for details).

Moreover, to test for general color biases in BR, we conducted a control experiment showing abstract gratings with the same colors from the colored objects of the color-diagnostic experiment (“gratings control”). This served as a baseline estimate for the conscious perception of the same color pairs in the complete absence of object information. To further control for object-color interactions, we performed a second control experiment with objects that do *not* have a color association, such as mugs (“nondiagnostic control”).

The study was separated into two sessions. In the first session, subjects would perform either the main color-diagnostic experiment or the nondiagnostic control experiment. In the second session, they would perform the gratings control experiment and the experiment that was not performed in the first session. This was counterbalanced across subjects. To familiarize participants with BR and ensure that subjects experienced perceptual alternations, they were shown gray images of basketballs and pumpkins for 120 s (not used in any of the later experiments). Moreover, participants performed a heterochromatic flicker task prior to each experiment to match the stimuli for subjective luminance for each stimulus separately.

Each experiment was divided into two parts: a sustained-rivalry and an onset-rivalry part. The sustained-rivalry part consisted of multiple runs (memory-color experiment: six runs, four trials each; nondiagnostic control: four runs, six trials each; gratings control: two runs, four trials each), where each trial lasted 90 s with an intertrial interval (ITI) of 10 s. The onset rivalry part consisted of a single run with several trials (memory-color experiment and nondiagnostic control: 96 trials; gratings control: 64 trials), where each trial lasted for at most 5 s with an ITI of 1 s. In the onset rivalry part, a trial ended as soon as the subject reported a dominant percept. At the end of each experiment, subjects were asked to fill out a questionnaire which asked for a subjective rating of their conscious perception during rivalry.

In the memory-color experiment, subjects were instructed to press one key during perception of the object in its congruent color and another key during perception of the object in its incongruent color. In the control experiments, subjects were instructed to press one key if they saw the object in red or blue and another in green or yellow. During piecemeal and mixed percepts, subjects were instructed not to press any key. Key assignments were counterbalanced across subjects.

### Stimuli generation and display

#### Memory-color experiment

**Figure 1:**
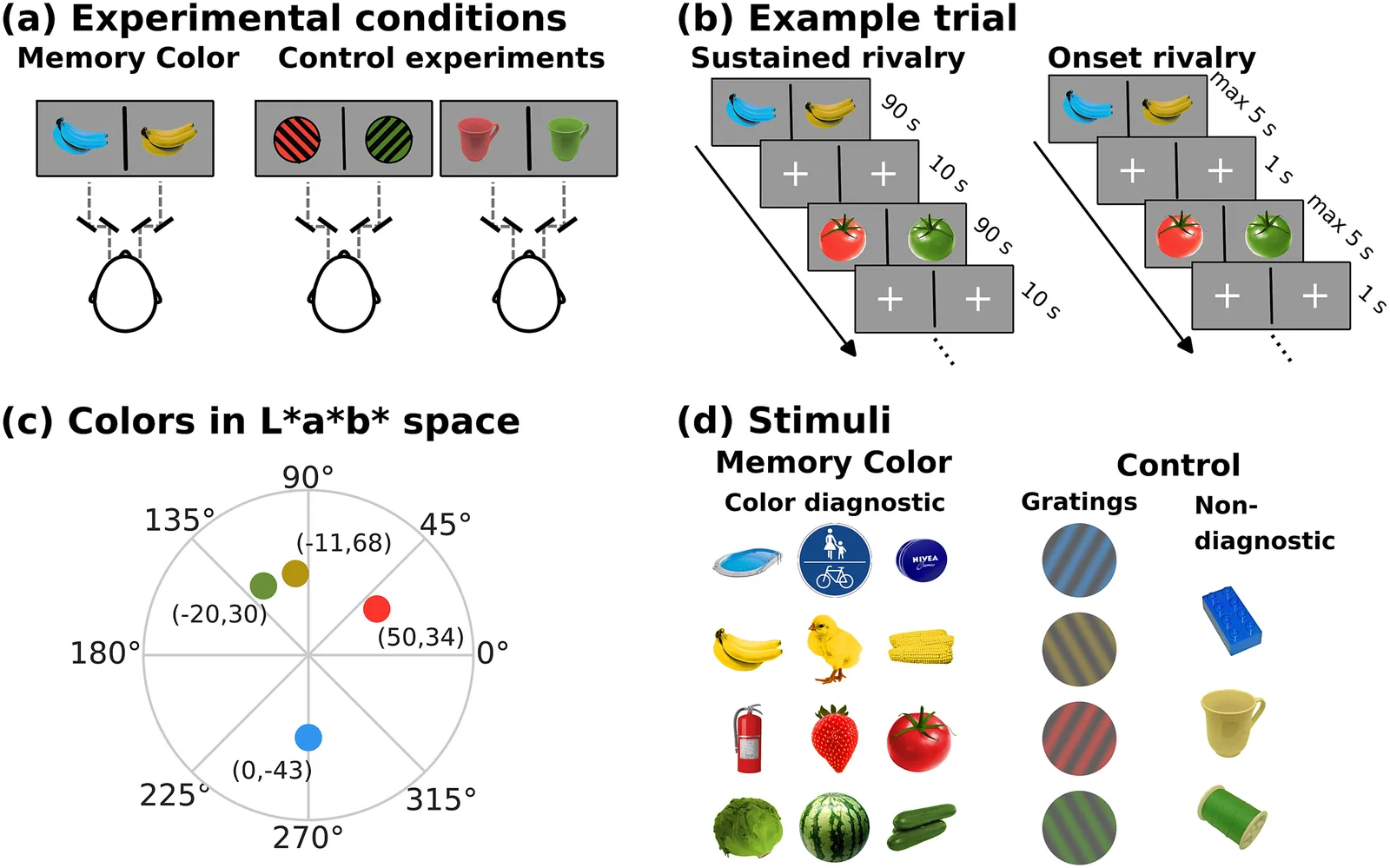
Experimental design. (a) Overview of the experimental BR conditions. The memory color experiment presented diagnostic objects in their congruent and incongruent color (such as a yellow and blue banana) to each eye separately. In the control experiments, we used the same colors as in the memory-color experiments but on neutral stimuli, such as colored gratings (first control) or nondiagnostic objects (second control). (b) Example trials of the sustained-rivalry (left) and onset rivalry (right) part of the memory-color experiment. During sustained rivalry, color-diagnostic objects were shown in congruent color to one eye and incongruent color to the other eye for 90 s with 10 s inter-trial interval. During onset rivalry, the same objects were shown either until subjects reported a percept or 5 s passed. (c) Mean hue angles for each color used in L^*^a^*^b^*^ space with their corresponding a^*^ and b^*^ values shown in parentheses. (d) Stimuli used in each BR experiments. For a detailed description of how these stimuli were preprocessed for the experiments, see also Supplementary section “Stimulus generation.”

In our main experiment, we used a set of 12 color-diagnostic objects, three per color. All objects were taken from Teichmann et al. (2020), except for a green watermelon and all blue objects that are public domain images taken from the internet (see Fig. 1d for all objects). The red, green, and yellow stimuli used have been shown to invoke an MCE in a recognition task (Teichmann et al. 2020). Stimuli were created such that confounding factors due to low-level visual differences, such as luminance or contrast, were minimized. In short, we extracted one hue for each of the four colors of the selected objects. We then projected these hues on equiluminant grayscale versions of the object images in order to create congruently and incongruently colored objects (see Supplementary section “Stimulus generation” and Supplementary Fig. S1 in the supplementary material for details). This procedure also ensured that incongruent hues on some objects corresponded exactly to congruent hues of other objects.

The stimuli (1.5°–2° of visual angle in size) were presented on a gray square of 3° of visual angle inside a Mondrian mask of 10° of visual angle (to aid binocular fusion). To increase mutually exclusive rivalry between images, individual stimuli were either rotated 10° to one another or flipped along the vertical axis (in case of vertical stimuli, such as a fire extinguisher).

#### Gratings control experiment

In a first control experiment, we tested for general color biases using colored gratings using the hues selected for the memory-color experiment. Gratings were shown rotated by +30° on the one and − 30° on the other eye. Stimulus size was 2.5° of visual angle, and the spatial frequency was 1.6 cycles/visual degree.

#### Non-color-diagnostic control experiment

In a second control experiment we used objects with no color association (Brodeur et al. 2010)—a mug, a lego block, and a thread spool—to control for object-color interactions independent of memory-color. Control stimuli were created the same way as the stimuli from our memory-color experiment, with the exception that we used the hues from the memory-color experiment to color equiluminant gray-scaled images of nondiagnostic objects. The three objects were presented in all colors and had the same dimensions as the objects in the memory-color experiment.

#### Post-experiment questionnaire

After each experiment, subjects were asked to fill out a short questionnaire. Each questionnaire asked for a subjective assessment of perceptual dominance during the sustained-rivalry and onset rivalry part. In the second control experiment using non-color-diagnostic objects, subjects were additionally asked to indicate whether they associate any color with the objects used. As expected, most participants did not report any color associations for the three objects used in the second control experiment (thread spool: 86%, mug: 90%, and lego block: 62%). Please note that we have questionnaire data from 20 subjects for all questions and that one subject failed to answer one of the questions (“Did you have the impression you perceived more time the diagnostic color or the nondiagnostic color?”).

### Data processing

For all data analyses, we removed mixed percepts from the sustained-rivalry data as well as all missed trials from the onset rivalry data (i.e. trials in which subjects did not press any button within the time window of 5 s). Furthermore, we assessed if data from any subject was unsuitable for data analysis. Exclusion criteria were an eye dominance of more than 70%, a median dominance duration shorter than 0.5 s and mixed percept durations of more than 70%. No subject had to be excluded. Finally, as the initial phase of rivalry is more susceptible to attentional biases (Mitchell et al. 2004, Chong et al. 2005, Alpers and Gerdes 2007), and might differ from sustained rivalry (Carter and Cavanagh 2007), we removed the first percept for the assessment of predominance ratio and median dominance duration. Please note that in this process some trials were excluded entirely, since for some subjects there were no switches in dominance at all in some trials (eight trials in four subjects, mostly perception of congruent red or yellow—see Supplementary Table S1 for details). Analyses of the data with or without the first percept did not affect the significance of the results.

### Data analysis

To investigate the MCE on conscious perception during BR, we extracted three measures from our data: *onset dominance, predominance, and median dominance duration*.

#### Onset dominance ratio

First, we were interested in the MCE on the very first percept after rivalry onset. For each color pair, we selected an arbitrary reference color: red as a reference color for the red-green and yellow as a reference color for the yellow-blue color pairs. We then calculated the onset dominance ratio (ODR) as the following ratio: 


\begin{align*} ODR=\left({N}_r-{N}_o\right)/\left({N}_r+{N}_o\right) \end{align*}

where *N_r_* and *N_o_* are the number of times reference and other color were reported as first percepts, respectively.

#### Predominance ratio

Furthermore, we were interested in how memory color affects the dominance *duration* of rivaling images. This measure thus quantifies the sustained biasing effect of object knowledge on conscious color perception. The predominance ratio (PR) was calculated as: 


\begin{align*} PR=\left({d}_r-{d}_o\right)/\left({d}_r+{d}_o\right) \end{align*}

where *d_r_* and *d_o_* are total durations of reference or other color across all trials.

#### Median dominance duration

Finally, the predominance ratio does not provide any information about how long each percept lasts. Hence, we compared median dominance durations (MDDs) for congruent and incongruent colors within objects (e.g. dominance duration for a yellow banana versus a blue banana), as well as across objects (e.g. dominance for a yellow banana versus a yellow nivea tin). We further calculated the difference in MDD for each color-pair (i.e. red-green and yellow-blue), separately per condition.

### Statistical inference

To assess the influence of an MCE in the main memory-color experiment, we compared each of the measures (ODR, PR, and MDD difference) where the reference color was the *congruent* color compared to where the reference color was the *incongruent* color, resulting in a 2 × 2 repeated-measures ANOVA (rmANOVA) with congruency (congruent/incongruent) and reference color (red/yellow) as factors. Please note that this approach controls for potential color biases because objects were presented in the same color pairs irrespective of whether the positive or the negative color was its associated memory color. Differences in MDD within and across objects were tested using paired *t*-tests. To test for general color biases in context of nondiagnostic objects and gratings, we performed one-sample *t*-tests on each of the measures using data from the control experiments using colored gratings and nondiagnostic objects. As these stimuli have no congruency, we only tested measures against zero. All *t*-tests were two-sided.

## Results

### Color-diagnostic objects

#### Onset dominance ratio

**Figure 2:**
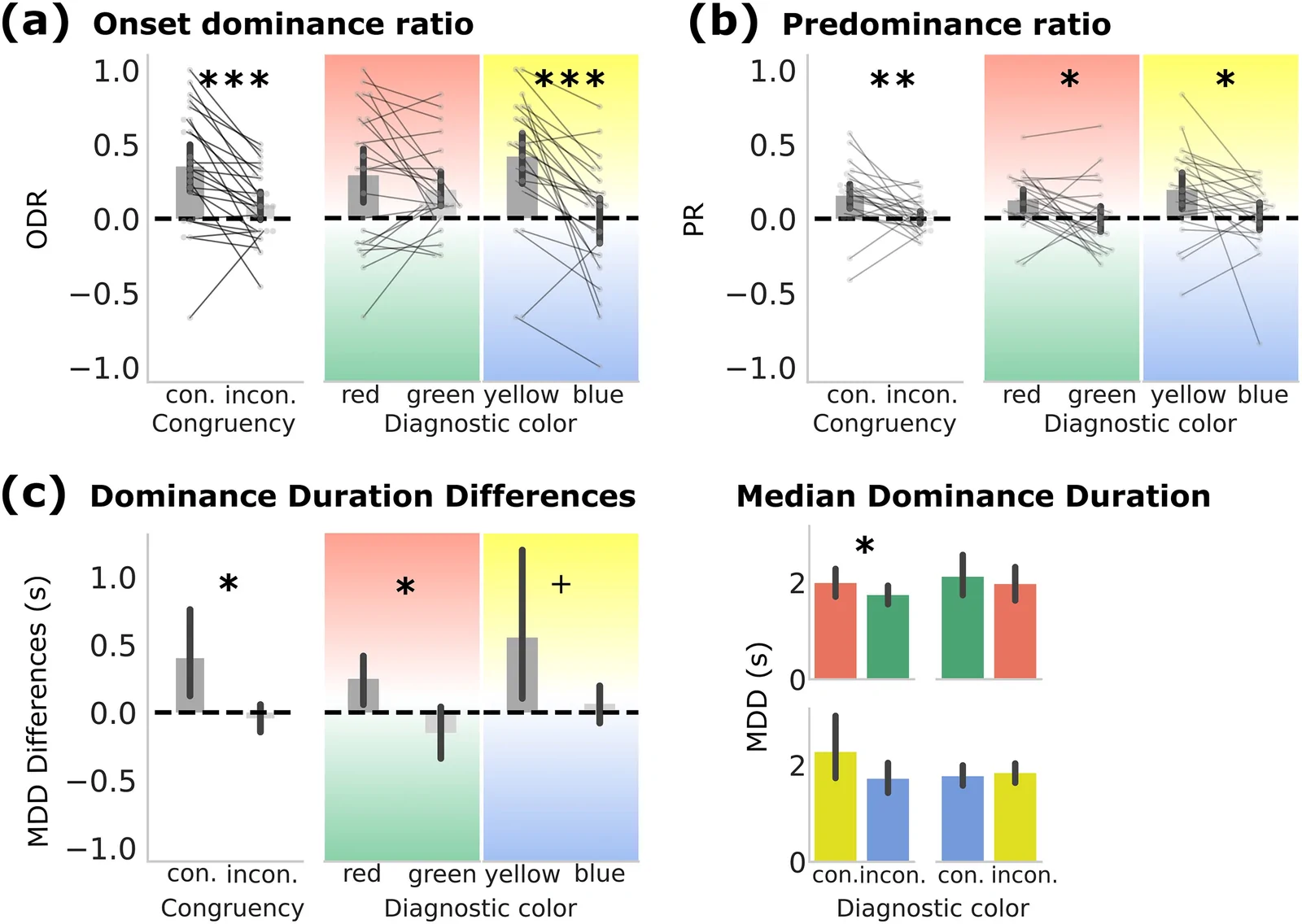
Results of the memory-color experiment. Shown are the results from paired comparisons of (a) onset dominance ratio (ODR), (b) predominance ratio (PR), and (c) difference in median dominance duration (MDD) averaged over all colors (left panels) and separately for each color pair (middle and right panels). (d) MDDs compared within objects (e.g. dominance for a red strawberry versus a green strawberry). All error bars depict 95% confidence intervals. ^+^  *P* < .1; ^*^, *P* < .05; ^**^, *P* < .01; ^***^, *P* < .001.

First, we tested whether memory-based object-color associations influence the first percept at rivalry onset. The rmANOVA of the ODR revealed a significant main effect for congruency, *F*(1,23) = 16.14, *P* = .001, eta^2^ = 0.097, and a significant interaction of congruency and reference color, *F*(1,23) = 13.5, *P* = .001, eta^2^ = 0.041, but no significant main effect for the reference color, *F*(1,23) = 0.19, *P* = .669, eta^2^ = 0.002. Post-hoc *t*-tests showed that the congruent color was reported more frequently, *t*(23) = 4.02, *P* = .001, Cohen’s *d*_avg_ = 0.8, 95% CI [0.13, 0.4]. This main effect of congruency was driven by the yellow-blue color pairs, *t*(23) = 5.24, *P* < .001, Cohen’s *d*_avg_ = 1, 95% CI [0.26, 0.59], but not the red-green pairs, *t*(23) = 1.28, *P* = .213, Cohen’s *d*_avg_ = 0.25, 95% CI [−0.06, 0.26] (see Fig. 2a).

#### Predominance ratio

The rmANOVA of the PR during sustained rivalry revealed a significant main effect for congruency, *F*(1,23) = 9.61, *P* = .005, eta^2^ = 0.091, but no significant main effect for reference color, *F*(1,23) = 0.95, *P* = .34, eta^2^ = 0.013, nor a significant interaction, *F*(1,23) = 0.17, *P* = .688, eta^2^ = 0.001. A post-hoc *t-*test revealed that objects were perceived longer in their congruent color than in their incongruent color, *t*(23) = 3.1, *P* = .005, Cohen’s *d*_avg_ = 0.88, 95% CI [0.05, 0.25] (see Fig. 2b).

#### Median dominance duration difference

Analyses of ODR and PR revealed that objects were perceived more often in their congruent color at rivalry onset and that objects were perceived for a longer time during sustained rivalry. Similarly, MDD difference values differed with respect to congruency, *F*(1,23) = 6.6, *P* = .017, eta^2^ = 0.071. There was no significant main effect for the reference color, *F*(1,23) = 2.19, *P* = .152, eta^2^ = 0.025, nor for the interaction *F*(1,23) = 0.09, *P* = .764, eta^2^ = 0.001. A post hoc *t-*test showed that dominance durations for objects in their congruent color were longer than in their incongruent color, *t*(23) = 2.57, *P* = .017, Cohen’s *d*_avg_ = 0.74, 95% CI [0.09, 0.8] (see Fig. 2c). Comparison of MDD within objects (e.g. yellow versus blue banana) revealed a significant difference for objects associated with red only, *t*(23) = 2.57, *P* = .017, Cohen’s *d*_avg_ = 0.39, 95% CI [0.05, 0.45] (see Fig. 2d). Detailed results for post-hoc comparisons can be found in Supplementary Tables S2 and S3.

Together, we find effects of congruency across all three measures of binocular rivalry that we analyzed.

### Control experiments

We further examined the same BR metrics in the absence of object knowledge but using identical color stimuli. In a first control experiment, we used colored gratings. Here, we observed that perceptual report of red gratings was significantly more frequent in all measures compared to green gratings (ODR: *t*(23) = 9.54, *P* < .001, Cohen’s *d*_avg_ = 1.95; PR: *t*(23) = 4.14, *P* < .001, Cohen’s *d*_avg_ = 0.84; MDD *t*(23) = 4.41, *P* < .001, Cohen’s *d* = 0.9). In contrast, we did not observe any significant bias toward blue or yellow gratings (ODR: *t*(23) = 1.2, *P* = .242, Cohen’s *d*_avg_ = 0.24; PR: *t*(23) = 1.05, *P* = .305, Cohen’s *d*_avg_ = 0.21; MDD: *t*(23) = 1.52, *P* = .143, Cohen’s *d*_avg_ = 0.31, see Supplementary Fig. S2). Analyses of a second control experiment using nondiagnostic objects yielded qualitatively similar results. For details, see supplementary results section *nondiagnostic objects* and Supplementary Fig. S2. Hence, both control experiments revealed a general bias toward red perception. Note that this bias cannot explain the preference for congruent colors observed in the memory-color experiment, which was mainly driven by yellow and green objects. If anything, the color bias would have worked against the main findings.

### Post-experiment questionnaire

Results of the post-experimental questionnaire reflected behavioral responses in the experiment. Subjects reported to have perceived objects more in their diagnostic color, *t*(18) = 2.96, *P* = .008, Cohen’s *d*_avg_ = 0.68. This was the case especially for red, *t*(19) = 4.47, *P* < .001, Cohen’s *d*_avg_ = 1, and yellow objects, *t*(19) = 3.48, *P* = .002, Cohen’s *d*_avg_ = 0.76. The ratings further reflected the general red bias found in the behavioral data with a strong bias toward red in both control experiments (gratings: *t*(19) = 3.24, *P* = .004, Cohen’s *d*_avg_ = 0.72; nondiagnostic objects: *t*(19) = 11.38, *P* < .001, Cohen’s *d*_avg_ = 2.54). No bias toward yellow or blue was found in neither control (grating: *t*(19) = 1.28, *P* = .217, Cohen’s *d*_avg_ = 0.29; nondiagnostic objects: *t*(19) = 0.43, *P* = .673, Cohen’s *d*_avg_ = 0.1).

## Discussion

In this study, we tested whether memory-based object-color associations can influence conscious perception during BR. To this end, we presented dichoptically color-diagnostic objects in their associated color to one eye and in an incongruent color to the other eye. We observed an MCE in all measures such that an object’s typical color determined which of two rivaling colors entered visual consciousness and for how long it remained dominant.

Our results suggest that memory-based associations affect spontaneous and automatic *conscious perception*. We observed that during ambiguous visual input a congruent object-color association is favored relative to an incongruent association in conscious perception. Our control experiments show that the results cannot be explained by low-level features or general color biases.

### Memory affecting perception or cognitive bias?

It has recently been argued that the visual system is “encapsulated” from higher-level cognition, and that (all) prior studies that report cases of top-down effects in perception—including memory color—can actually be explained by (or are susceptible to) a series of methodological and conceptual pitfalls (Firestone and Scholl 2016). The pitfalls often stem from conflating effects on perception with effects on *judgment* or *attentional processes*. For example, it has been suggested that memory color influences *judgments* and *responses* about color, but not the actual visual *appearance* (Valenti and Firestone 2019)—particularly in studies in which participants are asked to adjust the color of color-diagnostic objects to gray (as in Hansen et al. 2006, Olkkonen et al. 2008, Witzel et al. 2011).

In contrast, our experiment used BR, where participants simply reported the color that spontaneously and automatically entered their consciousness and is thus less susceptible to explanations based on post-perceptual processes. Unlike adjustment or comparison tasks that tried to show an MCE for *color appearance*, BR offers a more direct measure of *what color is* consciously perceived, a fundamental aspect of perceptual experience. This approach reduces the scope for judgment-based confounds, while providing a tool to measure memory influences on perception. Thus, although our results do not address whether memory color alters *color appearance*, they show a complementary perceptual effect, namely that memory-congruent colors dominate under ambiguity.

However, further non-perceptual factors and factors, which are not specific to memory color, could have contributed to our results. First, observers may have been more likely to attend to congruently colored objects. (Note, that this would not invalidate our conclusions but rather provide a mechanism for our effects.) Since attended stimuli typically dominate in BR (Dieter and Tadin 2011, Paffen and Alais 2011), particularly the first percept (Ooi and He 1999, Mitchell et al. 2004, Chong et al. 2005, Alpers and Gerdes 2007), any effect of memory color on perception may therefore not be direct but rather mediated by attentional processing. Such attentional mediation, however, is unlikely because incongruently colored objects are known to capture attention more (Cutler et al. 2024). It is hence more plausible that memory color directly influenced perception. Second, color congruency may have changed decision behavior (e.g. due to an attentional mechanism or directly) thereby influencing dominance reports via response hysteresis or criterion shifts. However, there is no reason to believe that such response biases skew the data favoring congruently colored objects during rivalry, and therefore account for our observations. Notably, the present results favor congruent percepts, while a prior scene study demonstrated dominance of incongruent percepts (Mudrik et al. 2011), consistent with increased performance in visual search for incongruently colored objects (Cutler et al. 2024). Future work could unequivocally prevent such post-perceptual response biases by employing no-report paradigms to investigate memory color effects on perception (Tsuchiya et al. 2015).

### Enhancement of expected stimuli and Bayesian inference

The observed MCE means that colors are more likely to enter visual consciousness if they match object-based expectations compared to when they do not. This observation is consistent with several studies that report a preference in BR for congruent and expected stimuli, such as upright compared to inverted faces (Engel 1956), predicted motion (Denison et al. 2011, Attarha and Moore 2015), known and newly learned cross-modal associations (Conrad et al. 2010, 2013, Chen et al. 2011, Lunghi et al. 2014, Einhäuser et al. 2017, Piazza et al. 2018), naturalistic stimuli (Baker and Graf 2009), familiar stimuli (Yu and Blake 1992), and percepts matching preceding mental imagery (Pearson et al. 2008). Beyond rivalry, this enhancement of congruent or expected stimuli also extends to other ambiguous paradigms, such as bistable structure-from-motion stimuli (cf. Gilroy and Blake 2004, Sterzer et al. 2008).

These convergent findings support the notion of perception as a Bayesian inferential process, where prior information (such as newly learned associations or visual history) is combined with sensory information to determine the most probable cause of the sensory inputs (Lee and Mumford 2003, Kersten et al. 2004, Friston 2005). In our study, objects in their congruent colors likely provide higher prior probabilities compared to incongruent colors, biasing perception in their favor. This aligns well with Bayesian models of BR (Hohwy et al. 2008) and perception in general (Clark 2013, Hohwy 2014, de Lange et al. 2018), suggesting that the visual system resolves ambiguity by favoring interpretations consistent with expectations.

However, this presents a paradox: novel and surprising stimuli are theoretically more informative for the visual system and convey new information for learning (Press et al. 2020), which is likely why color-diagnostic objects in incongruent colors capture attention (Cutler et al. 2024). As it happens, some BR experiments report enhanced dominance for incongruent objects in a scene (Mudrik et al. 2011) and unexpected images in a sequence (Denison et al. 2016), seemingly at odds with our findings and the Bayesian preference for priors. As novel and surprising stimuli are known to capture attention (Theeuwes 1992, 2010, Itti and Baldi 2005, Brockmole and Henderson 2008, Underwood et al. 2008, LaPointe and Milliken 2016), these conflicting results could be related to differences in attentional capture and allocation of attentional resources between experiments (Dieter and Tadin 2011). Moreover, Mudrik et al. (2011) and Denison et al. (2016) used complex naturalistic stimuli, in contrast to the simpler stimuli used in most studies showing an advantage in perception for the expected stimuli (e.g. Pearson et al. 2008, Denison et al. 2011, Attarha and Moore 2015). Thus, the direction of perceptual benefit—favoring expectation or novelty—may additionally depend on task demands, stimulus complexity, and processing stage. For example, Mudrik et al. (2011) observed a preference for the unexpected/incongruent percept when scene perception aided ambiguous object perception (favoring novelty), whereas we observe a preference for expected/congruent percept when object perception aided ambiguous feature perception (favoring expectation). These differences are likely related to where exactly BR is resolved in the brain (see also Lawler and Silver 2023), and how predictive signals of different complexity interact with attentional processes therein (Dieter and Tadin 2011).

Although congruently colored objects were more dominant than incongruently colored objects on all three BR measures, the effect of congruency on onset rivalry differed between the red-green and blue-yellow color pairs. It is known that memory color effects differ not only across objects and colors but also across experimental procedures (Tanaka and Presnell 1999, Olkkonen et al. 2008, Witzel et al. 2011). Witzel et al. (2011) reported stronger effects along the daylight axis, which roughly matches our yellow-blue pair, suggesting that observers rely more on memory when perceiving object color because sensory inputs vary more strongly along this direction over the course of day. Furthermore, our color pairs likely engaged different cone mechanisms, L–M *versus* S, which differ in cortical tuning sensitivity (e.g. Engel 1999, Liu and Wandell 2005). Our observations may thus reflect generic chromatic differences unrelated to non-chromatic object properties and object knowledge. Additionally, because objects are typically warmer than their backgrounds (Rosenthal et al. 2018), blue objects may appear less common than others, potentially affecting expectations and diminish recognizability. Since variability in MCE likely depends on factors such as object frequency, familiarity, strength of color association, etc. as well as on neural mechanisms, research is needed to disentangle these.

### How memory color biases binocular rivalry

In BR, competition can occur at multiple levels of the visual pathway (Blake and Logothetis 2002, Tong et al. 2006). For example, neuroimaging shows eye-specific rivalry in V1 (Haynes et al. 2005; Qian et al. 2026) and in ventral color (Kim et al. 2020) and face and place areas (Tong et al. 1998). Moreover, neuroimaging (Zaretskaya et al. 2010, Weilnhammer et al. 2013, 2021), electrophysiology (Panagiotaropoulos et al. 2012, Kapoor et al. 2022), and TMS experiments (Carmel et al. 2010, Kanai et al. 2010, Zaretskaya et al. 2010, Weilnhammer et al. 2021, Schauer et al. 2024) have presented compelling evidence for the role of frontal and parietal areas in resolving and modulation perceptual ambiguity (see Brascamp et al. 2018 for a review). Rivalry activity in visual areas may thus reflect modulatory feedback signals from higher frontal and parietal areas (Maier et al. 2008, Grassi et al. 2016a, De Jong et al. 2020, Qian et al. 2026). This is compatible with “predictive coding” models implementing Bayesian inference, which see top-down memory-based predictive signals being fed-back to lower-level sensory cortices to be compared with bottom-up input (Mumford 1992, Rao and Ballard 1999, Lee and Mumford 2003, Friston 2005) and neuroimaging evidence using ambiguous stimuli (Murray et al. 2002, De-Wit et al. 2012, Zaretskaya et al. 2013, Grassi et al. 2016b, 2017, 2018).

Our behavioral observation of a memory-color effect enhancing input based on prior knowledge in BR is in line with existing evidence suggesting top-down modulation of rivalry signals in visual cortices. Moreover, our results are supported by further neuroimaging reports of neural information in visual areas signaling memory color (Bannert and Bartels 2013, Vandenbroucke et al. 2016) and surprise based on unexpected naturalistic color changes (i.e. color changing magic tricks; Plikat et al. 2025). Hence, our results show that in cases of ambiguous visual stimulation, prior knowledge influences which stimulus enters conscious perception, consistent with the existence of top-down signals in the visual cortex.

### On cognitive penetrability of perception

We observe that knowledge of an object’s typical color influences conscious perception. But is this a case of cognitive penetrability? This depends first, on how one draws the border (if at all) between cognition and perception (Cermeño-Aínsa 2020, Vetter et al. 2024), second, on the nature of object-color associations (cognitive versus perceptual), and finally, on the mechanism by which these associations potentially affect color perception (e.g. visual cortex may be involuntarily involved/reactivated when retrieving color knowledge via color imagery, Macpherson 2012). Crucially, the present (and all prior) experiments do not allow us to differentiate between more conceptual, semantic color knowledge (e.g. knowing that bananas are yellow) and perceptual priors reflecting learned object-color regularities (i.e. based on exposure to yellow bananas). If object-color associations are stored as perceptual priors within the visual system, then the purported memory color effect may not be a clear case of cognitive penetrability as it could simply reflect intra-perceptual processes (Block 2023). We cannot distinguish between these two cases.

Yet, our study does show, first, an effect on perception (potentially in contrast to prior memory color studies). Second, we observe this using a paradigm with minimal task demands and reduced judgment-based confounds, suggesting an automatic and spontaneous process. Finally, the congruency effects observed here necessarily presuppose object recognition and a comparison with stored memory representations.

Prior evidence has shown that object recognition and comparison with stored representations occur after simple color processing (at around 250 ms, cf. Teichmann et al. 2020). Moreover, evidence from semantic dementia (Rogers et al. 2007), brain lesions (Miceli et al. 2001, Bouvier and Engel 2006, Stasenko et al. 2014), and neuroimaging (Chao and Martin 1999, Hsu et al. 2015, Price et al. 2017) dissociate color knowledge from color perception and suggest that object-color associations are stored in areas of the temporal cortex more anterior to those involved in color perception. These are regions at the boundary between visual and higher-level cognitive processing.

Hence, while our results do not show that “belief-like” cognitive states directly influence perception, they do indicate that color experience can be biased in a top-down manner by memory object representations beyond simple color-processing stages. This shows that conscious color perception is object-dependent, while leaving the question if it is sensitive to higher-level conceptual knowledge (Deroy 2013).

## Conclusion

Here, we combined memory-color with BR to show that when confronted with ambiguous color stimuli, prior knowledge about an object’s typical color influences conscious perception. We observed a significant influence of memory-color in BR, favoring expected colors when viewing color-diagnostic objects, consistent with Bayesian models of BR. These results cannot be explained by low-level differences or general color biases and are difficult to attribute to residual differences in attention or judgment. Instead, we argue that the effects reflect a genuine top-down effect of knowledge in perception.

## Supplementary Material

BR_and_Memory_Color_Sup_niag055

## Data Availability

Data, experimental code, and analysis code are available at https://osf.io/hdfw4/.
