# Supplementary material for: Memory color influences conscious object perception
Source: Neurosci Conscious. 2026 Sep 2;2026(1):niag055. doi: 10.1093/nc/niag055 (PMC13537532; doi:10.1093/nc/niag055)
Supplement: Supplementary file 1 [file br_and_memory_color_sup_niag055.pdf]

Supplementary Material: Memory color influences conscious object perception

Vincent Plikat[1,2,3], Pablo R. Grassi[1,2,3], Michael M. Bannert[1,2,3], Andreas Bartels[1,2,3]
[1] Vision and Cognition Lab, Department of Psychology, University of Tübingen, Tübingen, Germany
[2] Centre for Integrative Neuroscience, Tübingen, Germany
[3] Max-Planck Institute for Biological Cybernetics, Tübingen, Germany

Content

Supplementary Methods 2
  Stimulus generation 2
  Data preprocessing: first percept extraction 3
Supplementary Results 4
  MDD within and across color diagnostic objects 4
  Second control experiment: Non-diagnostic objects 5
References 5

Supplementary Methods

Stimulus generation

Binocular rivalry (BR) is modulated by low-level visual features, such as contrast, luminance and spatial frequency (Brascamp et al., 2015). To control for these differences, we generated stimuli that were matched in low-level visual features and only differed in color. The generation of stimuli was done by following these steps: (1) first, for each image we defined a color and a luminance mask. These masks excluded pixels different from the diagnostic color (e.g., yellow seeds on a red strawberry) and pixels close to extreme luminance (i.e., white and black). (2) Then, we extracted, for each object, the median hue angle in L*a*b* space using the color mask from step (1) and calculated the mean angle for each cardinal color (red= 34° (a=50,b=34), green= 123° (a=-20,b=30), yellow=99° (a=-11,b=68), blue=270° (a=0,b=-34)). (3) Then, we made equiluminant grayscaled images from original stimuli (mean luminance within each image was 0.54) and projected median hue values once for the congruent color (e.g., green cucumbers) and once the incongruent color (e.g., red on cucumbers) using the color and luminance masks. All pixels within the masks were multiplied with the monitor's gamma function. (4) Finally, to make stimuli subjectively equiluminant, each subject performed a heterochromatic flicker task with objects in their congruent and incongruent color. In this task, all pixels within the color and luminance mask were scrambled and reassembled as a square. The squares consisting of pixels in congruent and incongruent color alternated each frame, resulting in a flickering percept, as long as these pixels were *subjectively* different in luminance. Subjects changed the luminance of the pixels until the flickering was minimized. Please note that during the heterochromatic flicker tasks pixels were multiplied with the monitor's *inverted* gamma function to preserve the contrast within an image.

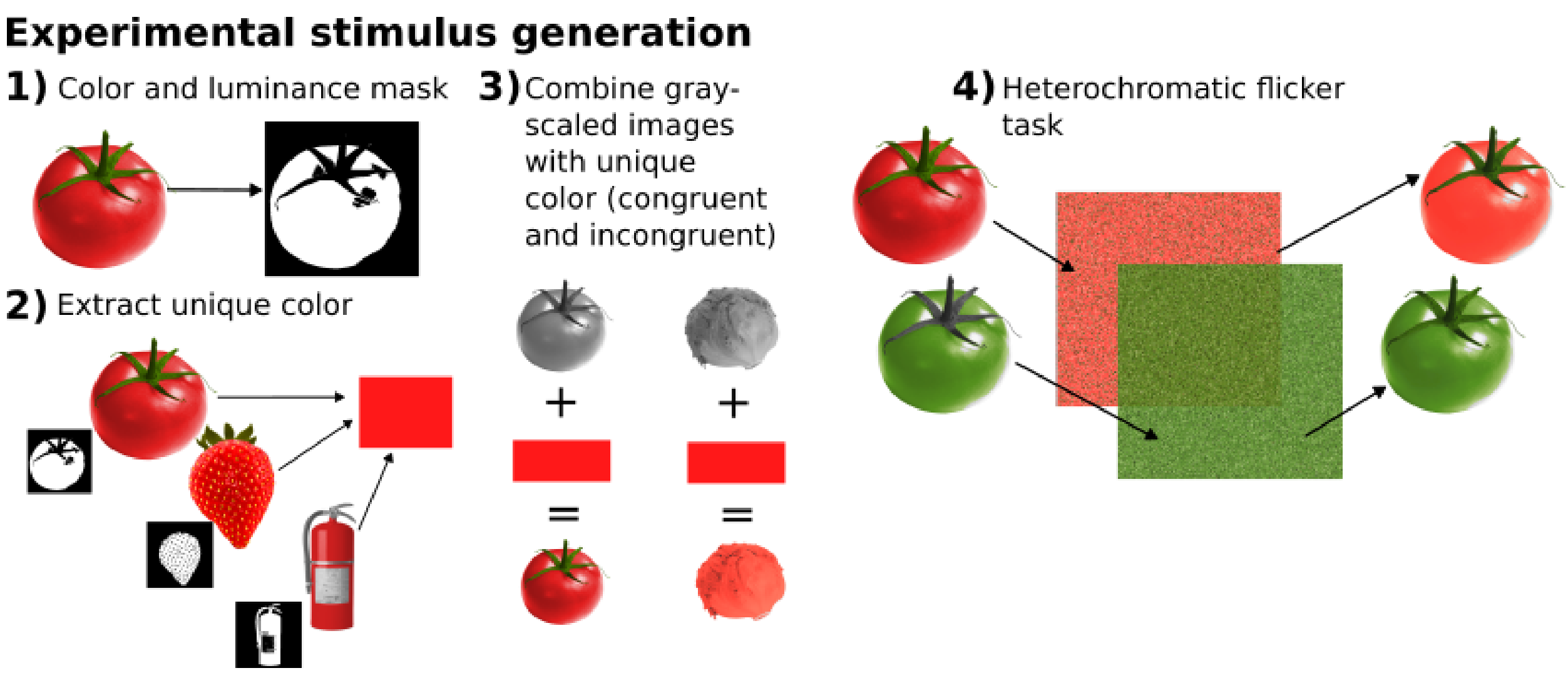

Fig. S1: **Experimental stimulus generation.** The figure shows a simplified explanation of how stimuli were generated. **1)** For each image a combined color and luminance mask was created. **2)** Then, for all diagnostic objects within one color, we applied the masks to extract one representative hue (in L*a*b* space), which was then **3)** applied to luminance-matched grayscale images such that the color was either congruent (e.g., red on a tomato) or incongruent (red on a lettuce). In a final step **4)** subjects performed a heterochromatic flicker task to make each image subjectively equiluminant.

Data preprocessing: first percept extraction

To correctly assess median dominance duration (MDD) and predominance in the sustained rivalry part of our experiments we excluded the first dominant percept of each trial. In a few cases, this led to the loss of entire trial data as in these there were no switches. Below, we provide a detailed list showing for which subject, object and trial the first and only dominant percept lasted.

Table S1.
Excluded trials due to missing percept alternations

SUBJECT	OBJECT	DURATION (S)	PERCEIVED COLOR
**SUB-02**	Cucumbers	88	Incongruent (red)
**SUB-02**	Fire extinguisher	88	Congruent (red)
**SUB-02**	Fire extinguisher	89	Congruent (red)
**SUB-07**	Fire extinguisher	89	Congruent (red)
**SUB-07**	Pool	4	Incongruent (yellow)
**SUB-07**	Tomato	89	Congruent (red)
**SUB-14**	Bananas	71	Congruent (yellow)
**SUB-19**	Corn	1	Congruent (yellow)

Note: Information of excluded trials with only one dominant percept. In most cases (6 out of 8) participants perceived the object exclusively in its congruent color throughout the trial.

Supplementary Results

MDD within and across color diagnostic objects

Results from two-sided paired t-tests for MDD in the congruent compared to the incongruent condition within objects (e.g., MDD for red on a strawberry compared to green on a strawberry) and across objects (e.g., MDD for red on a strawberry compared to red on a lettuce) are shown in tables S2 and S3, respectively.

Table S2.
Comparison of MDD within objects

Color	T-value (df=23)	p-value	Cohen's d_{avg}	95% CI
**Red**	2.566	**0.017**	0.389	0.05 - 0.45
**Green**	1.495	0.149	0.152	-0.06 - 0.36
**Yellow**	1.869	0.074	0.425	-0.06 - 1.17
**Blue**	-0.843	0.408	0.121	-0.22 - 0.09

Note: Paired t-tests (two-sided) for MDDs of the congruent and the incongruent color within objects.

Table S3.
Comparison of MDD across objects

Color	T-value (df=23)	p-value	Cohen's d_{avg}	95% CI
**Red**	0.222	0.826	0.025	-0.17 - 0.21
**Green**	2.286	**0.032**	0.446	0.04 - 0.72
**Yellow**	1.683	0.106	0.355	-0.10 - 0.97
**Blue**	0.393	0.698	0.077	-0.22 - 0.33

Note: Paired t-tests (two-sided) for MDDs of the congruent and the incongruent color across objects.

Second control experiment: Non-diagnostic objects

Data analysis of the second control experiment (using non-diagnostic colors) revealed consistent results across our three dependent variables: onset dominance ratio (ODR), predominance (PR) and difference in median dominance duration (MDD). We observed a general bias towards red (compared to green) in all measures (ODR: $t(23) = 6.244$, $p < .001$, Cohen's $d_{avg} = 1.275$; PR: $t(23) = 6.371$, $p < .001$, Cohen's $d_{avg} = 1.3$; MDD: $t(23) = 3.960$, $p = .001$, Cohen's $d_{avg} = 0.808$). No such an effect was observed when comparing blue- and yellow colored non-diagnostic objects (ODR: $t(23) = 0.67$, $p = .509$, Cohen's $d_{avg} = 0.098$; PR: $t(23) = -0.294$, $p = .771$, Cohen's $d_{avg} = 0.06$; MDD: $t(23) = -0.635$, $p = .532$, Cohen's $d_{avg} = 0.130$, see Supplementary Figure S2).

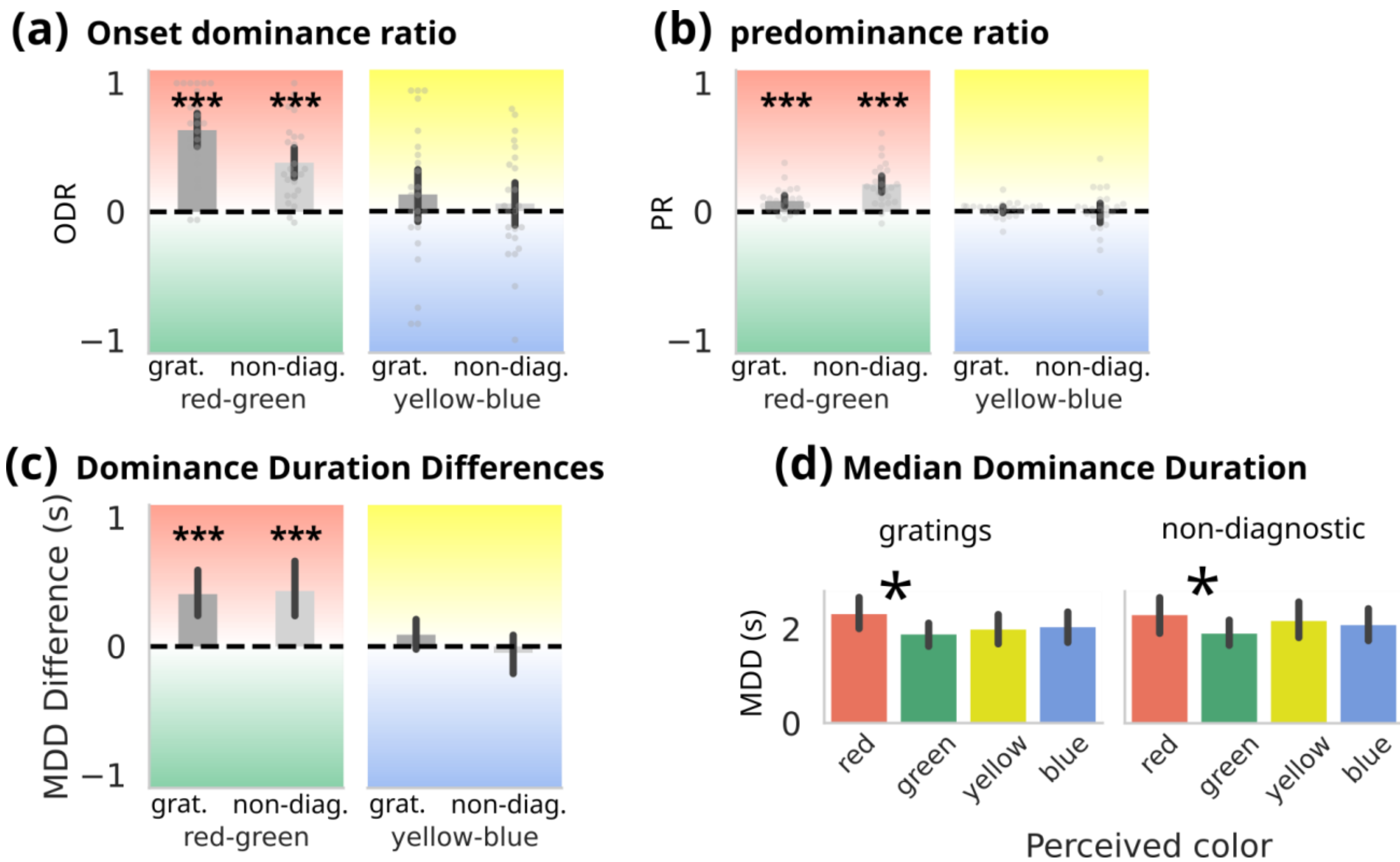

Figure S2: Results for the first control (using gratings) and second control (using non-diagnostic objects) experiments. **(a)**, **(b)** and **(c)** show onset dominance (ODR), predominance ratio (PR) and difference in MDD with respect to the reference colors (red and yellow) for both control experiments. **(d)** shows the MDD for each color in the first (gratings; left) and second control experiment (non-diagnostic objects; right). Grat: gratings (first control); non-diag.: non-diagnostic objects (second control). All error bars depict 95% confidence intervals. * $p < .05$; **, $p < .01$; ***, $p < .001$.

References

Brascamp, J. W., Klink, P. C., & Levelt, W. J. M. (2015). The “laws” of binocular rivalry: 50 years of Levelt’s propositions. *Vision Research*, *109*(Part A), 20–37. https://doi.org/10.1016/j.visres.2015.02.019
